# The Clinical Assessment in the Legal Field: An Empirical Study of Bias and Limitations in Forensic Expertise

**DOI:** 10.3389/fpsyg.2015.01831

**Published:** 2015-11-30

**Authors:** Antonio Iudici, Alessandro Salvini, Elena Faccio, Gianluca Castelnuovo

**Affiliations:** ^1^Department of Philosophy, Sociology, Education and Applied Psychology, University of PadovaPadova, Italy; ^2^Istituto di Psicologia e PsicoterapiaPadova, Italy; ^3^Psychology Research Laboratory, Istituto Auxologico Italiano IRCCS, Ospedale San GiuseppeVerbania, Italy; ^4^Department of Psychology, Catholic University of MilanMilan, Italy

**Keywords:** assestment, clinical trials as topic, bias, forensic psychiatry, forensic psychology, systematic error

## Abstract

According to the literature, psychological assessment in forensic contexts is one of the most controversial application areas for clinical psychology. This paper presents a review of systematic judgment errors in the forensic field. Forty-six psychological reports written by psychologists, court consultants, have been analyzed with content analysis to identify typical judgment errors related to the following areas: (a) distortions in the attribution of causality, (b) inferential errors, and (c) epistemological inconsistencies. Results indicated that systematic errors of judgment, usually referred also as “the man in the street,” are widely present in the forensic evaluations of specialist consultants. Clinical and practical implications are taken into account. This article could lead to significant benefits for clinical psychologists who want to deal with this sensitive issue and are interested in improving the quality of their contribution to the justice system.

## Introduction

This study refers to the application of clinical psychology in the forensic area (“forensic psychology”), which focuses on the interface between the justice system and mental health evaluation. A forensic psychologist, an expert in appropriately interacting with judges, attorneys and other legal professionals, is responsible to testify in court, reformulating psychological findings into the legal language of the courtroom and providing information to legal personnel in a form that can be understood (Melton et al., 1997). His/her competency is fundamental since the judge usually expected to educate himself on facts that may need additional interpretation and information by technical disciplines; in fact, the judge himself, an expert in legal matters, cannot go beyond his personal experience and knowledge and is inclined to use categories of common sense for personality evaluations (Heilbrun, 2001; Heilbrun et al., 2004). The main roles of a psychologist in the court system are eightfold: Evaluation of possible malingering, Assessment of mental state for insanity plea, Competency to stand trial, Prediction of violence and assessment of risk, Evaluation of child custody in divorce, Assessment of personal injury, Interpretation of polygraph data, and Specialized forensic personality assessment (Otto and Heilbrun, 2002; DeClue, 2005). “Questions” asked by the court of the forensic psychologist are generally related to a legal question rather than to a psychological one. As an example, the judge may appoint a forensic psychologist to assess the state of mind of the defendant at the time of the offense. Since the “question” is posed in legal terms (the responsibility for the crime), the forensic psychologist is called to translate psychological information (about the sanity or insanity of the person) into a legal framework. The forensic psychologist is thus invested with a great responsibility in providing the assessment information, including the diagnosis, treatment and any other information the judge requests, such as information regarding mitigating factors, and evaluation of witness credibility. Forensic psychology also involves training and evaluating police or other law enforcement personnel, providing law enforcement with criminal profiles and working in other ways with police departments. That is why he/she needs to be an expert advisor who can translate the words in the judge’s question into common sense technical-scientific constructs on which to base the assessment. The lexical formulations with which the judge indicates the scope and limitations of the investigation have been entrusted to an expert to create conventional “questions.” Just the interdisciplinary character of the matter can give rise to some criticalities in the role of the advisory forensic psychologist, particularly with respect to the complexity of applying psychological knowledge in such a different system as that of the Law (Nicholson and Norwood, 2000; Goldstein, 2003, 2006). The fact that forensic psychology is a relatively recent development that made its appearance in the universities in the late 1960s (Ogloff, 2002; Kovera et al., 2003) explains why there is no precise mode of action for the advising and assessment needs of the judge, but cannot constitute an excuse in the search for a rigorous definition of the plan of investigation (Drogin, 2001; Drogin and Barrett, 2003). The situation is delicate because the forensic field differs from many other areas (Rogers, 2000). For example, in a clinical field, a “inadeguate assessment” can be corrected afterward by psychologists or by others, but within forensics, the technical advice cannot be modified subsequently, and can therefore mislead the judge, with serious consequences for the freedom or the health of a third party (Greenberg and Shuman, 1997; Strasburger et al., 1997; Wiener et al., 2002). From this we can understand the foregone conclusion of how important it is to have a strong commitment to the epistemological framework, and be explicitly aware of the assumptions and expectations underpinning the methods used (Walker and Woody, 2011; McAuliff and Bornstein, 2012; Iudici and Renzi, 2015a).

## Legal Sciences and Psychological Sciences

The diagnostic evaluation in the forensic field, therefore, reflects the need to combine skills from different disciplines, especially the sciences of the psyche and the science of law (Drogin, 2007; Drogin et al., 2011). From the first, recognized as the referring science, come theories, paradigms, methods and tools, from the second, assumed to be just referring of context, comes a normative frame within which the forensic advisor may work, defining the role and tasks (Wrightsman, 1999; De Leo and Patrizi, 2002; Grisso, 2003; Grisso and Vincent, 2005; Patrizi, 2012). Sometimes the evaluation results tend to subordinate the former to the latter, accepting the configuration of the “events” as they predefine the code or the jurisprudence. In turn, the judge is called to pronounce on “facts” legally configured as the law (and implicitly, common sense) imposes. Nevertheless, as the judge cannot exceed his/her personal experience, some technical aid is required from the experts; however, this aid is bound to remain within its epistemic paradigm, and in fact, since the question to which the advisor reponds is legally codified, configured and preordained by the codes (and law) (Lind and Tyler, 1988; Boccaccini et al., 2002). The request, however, is guided by the implications of common sense (“ingenuous theories of mind and personality,” Bruner and Tagiuri, 1954) more than from coherent applications using the methods of the psychological science. For example, the advisor may be invited to assess a defendant on the understanding of intent, on the social danger, on the parenting skills, or on the competence to stand trial.

Since these expressions are borrowed from the legal or common language, the requests are not always the object’s employer. Terms like “real motivation,” “in personological characteristics,” “personality of the child” can appear clear under the lexical profile or by the common meaning, but are not rigorous in terms of scientific construct, that should always be specified; otherwise you may incur an improper reification. For details, consult Iudici and Verdecchia (2015b) and Iudici et al. (2015).

The scientific constructs require precise epistemological clarification, and therefore the skill to distinguish the discourse of the “man in the street” for their references of knowledge. This explains the demand for specialist opinion that goes beyond the common experience (Dalby, 1997; Gudjonsson and Haward, 1998).

There is no doubt that it is hard work to convert scientific knowledge into the methods and the criteria of “objective” (Drogin et al., 2011). For example, a request might ask the adviser, “What are the causes that lead the child to reject the meeting with the non-custodial parent?” The advisor must transform their own interpretations, suppositions, hypotheses, in “certain cases,” and is required to establish to whom the responsibility of such refusal is imputed (transformation of the cause in guilt, in the interpretation of causation, and reduction of a semeiotic interactive process in a linear determinism). In this situation, the opinion of the technician becomes a “judgment,” legally usable. The difficulty is to integrate two different epistemic levels to merge the requirements of the “legal truth” with the criteria of the scientific demonstration (Salvini et al., 2008). Also from a cognitive point of view, the process “constative” (facts) and “assessment” (constructs) develop different modes of reasoning, which, if they overlap, can lead to errors of attribution, which we shall discuss in the following paragraph.

## The Systematic Errors of Judgment

Forensic psychiatrists, psychologists and criminologists, despite the diversity of professional trainings and skills, traditionally share a similar purpose, strongly oriented to meet the judge’s expectations, translated into the formality of the put “question” (Shapiro, 1991). In many cases, the question focuses on the *personality construct*, and the consultant is asked to investigate, describe and explain the way of being and acting of people whose behaviors have acquired or may acquire a legal significance.

The generative matrix, and the focal point of this conceptual system is the “forensic assessment,” which intersects, as already observed, the description, explanation, evaluation, and argumentation (Cervone and Peake, 1986; Pohl, 2004).

The present research will use some knowledge from the “psychology of the attribution,” an area in which researchers have studied how the formation of judgments, expressed with an evaluative scope, explanatory and predictive, can be influenced by the underlying processes of assessment, like inferences, attributions of cause and its heuristic (Chapman and Johnson, 2002; Englich et al., 2006; Eerland and Rassin, 2012).

Processes that the cognitive psychologists and the social psychologists have dedicated themselves to understanding; and perhaps for this reason they have been extraneous to the knowledge of forensic and clinical psychology and psychiatry. The cognitive processes by which people judge others, make decisions, explain the causes of behavior, assess situations, use persuasive arguments, and implement reasoning, is a research area that has produced several important results. To try to explain how this can favor “systematic errors of judgment” is the focus on which it seems appropriate to dwell (Marshall and Alison, 2007; Bollingmo et al., 2009).

### Limitations and Risks of Forensic Expertise

If in the past the knowledge resources related to the preparation of an appraisal were limited, today many scholars have researched the limits of expert reports (Saks and Koehler, 2005) and defined guidelines for improving the work of the forensic technicians (Committee on Ethical Guidelines for Forensic Psychologists, 1991; American Psychological Association [APA], 2013).

There are several studies that highlight how experts in forensics might contaminate their own appraisal activities through bias, both historically (Miller, 1984; Stoel et al., 2014) and in the recent past (Proia, 2005; Budowle et al., 2009; Page et al., 2012).

Researchers’ efforts often focus on different themes, for example the risk of violence in adolescents, violent individuals, in cases of danger, in the sexual (Herman, 2005.) and in post-traumatic stress disorder (Grisso and Vincent, 2005). Many studies have shown how the main problem is the non-distinction between legal and clinical data [(A discovery generally stood out in four of the six studies (Greenberg and Shuman, 1997; Christy et al., 2004)]. Examiners often reported significant clinical data, and the legal matter has often been addressed, but the reports often do not actually identify the reasoning of the examiner on the connection between the clinical data and the opinion of the examiner on the relevant legally deficient candidate. In other words, he could not explain how their data were related to their opinions or the logic that connected them. Thomas Grisso (2003) has systematized a number of issues involved in the development of expertise, particularly in creating interpretations and opinions that are not contradictable, the confusion between data and interpretations, and problems related to the language used, particularly in the presence of category values.

## Design Study

The first part of the research was carried out in the following databases: Google Scholar and Scopus. The work dealt with analyzing some expert reports commissioned by a judge as part of civil proceedings. The research was divided into two objectives. The first objective aimed to identify the errors of judgment applied to the field in the forensic international literature. The etymology of the term “error” refers to the concept of “a way to deviate from established”: here the error is understood just as a divergence from the objectives that the forensic consultant intends to pursue. For example, if the aim is to provide a technical assessment, then judgment distortions do not have to be produced. If it is intended to follow a logical reasoning, the paths that go from the premises to the conclusions must not divert in comparison to the criteria of the schemes of logical reasoning. If the objective is to provide objective assessments, there should be no moral or value judgments. The criteria used in the identification of expertise was the surveyor mandate, or specifically the request by the court for “observation activities” and “psychological assessment of the personality structure of the parents.” All reports were compiled by a technical office adviser, or by consultants registered in the specific register of each Court. At the end of the study, only those relationships performed by professional psychologists were considered. The reports identified relate to the period 2010–2013. The eligibility criteria were: errors of attributions in forensic field, judgment systematic errors in forensic field, bias in the attribution of causality in forensic field, inferential errors in forensic field, epistemological inconsistencies in forensic field.

## Methods

The purpose of highlighting the possible errors was initially pursued by developing research in the social psychological literature, juridical and clinical, to identify possible errors and contextualize in reference to the forensic field. The examples are taken from analysis of 46 forensic relations. The work was carried out according to a method of qualitative analysis (Flick, 2008) and the procedure adopted is one of those described by Tuzzi (2003) for the analysis of the content. Specifically, we proceeded according to a “classic” approach with manual encoding of the texts (Miles and Huberman, 1984; Miles and Huberman, 1994; Denzin and Lincoln, 2005; Maxwell, 2005). The clinical consultants who made their documents available were recruited by the authors of the research. They signed an informed consent form about the design and purpose of the research. All relations were analyzed by two independent judges into 17 mutually exclusive categories, corresponding to the systematic judgment errors considered on the basis of the analysis of semantic content. The level of inter-rater agreement, measured by Cohen’s Kappa coefficient is 0.93. The categories were predefined by the researchers in accordance with the literature. Then the frequencies and percentages and descriptive statistics were calculated (minimum, maximum, mean, standard deviation) and graphs were created representing the total number of errors for each area and the average number of total errors by area.

## Results

Regarding the first objective, the following errors have been identified, grouped in the following areas.

**FIGURE 1:**
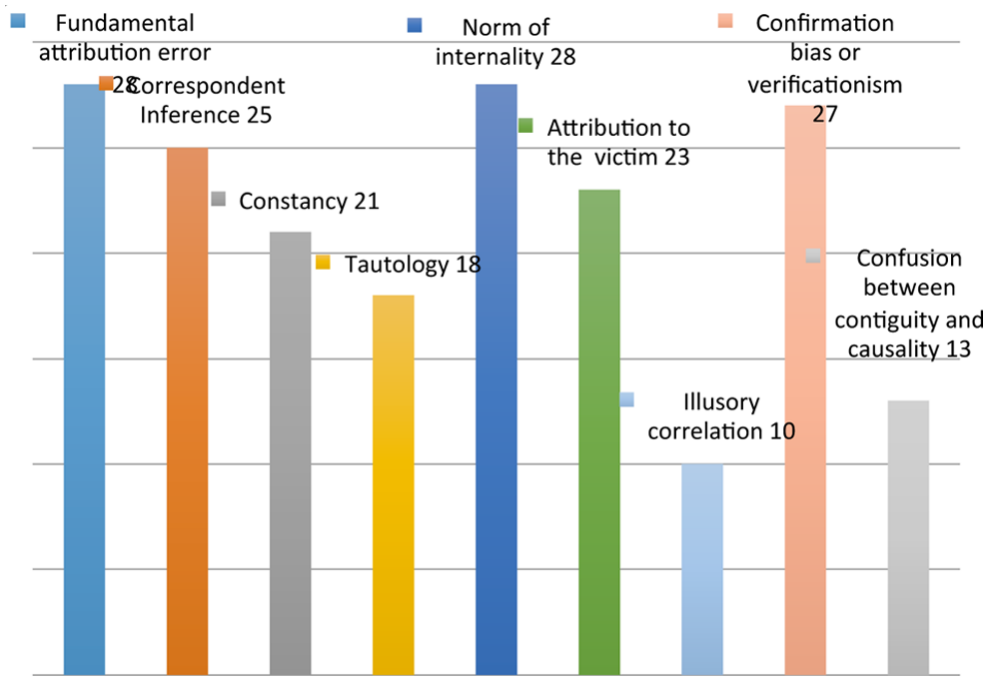
**Attribution errors**.

The errors (Appendix 1) were referable to three areas:

(a) The area of distortions in the attribution of causality, or errors that are encountered when investigating the causes and responsibility of the behavior, without considering the intentions of the person and the context (an example is the known one “fundamental error of attribution”);

- Fundamental attribution error (Heider, 1958; Ross, 1977)
- Correspondent inference (Jones and Davis, 1965)
- Constancy (Mischel, 1993)
- Tautology (Hewstone et al., 1996)
- Norm of internality (Beauvois and Dubois, 1988; Dubois, 2003)
- Attribution to the victim (Lerner, 1980; Lerner and Goldberg, 1999)
- Illusory correlation (Chapman, 1967; Tversky and Kahneman, 1974; Hamilton and Gifford, 1976)
- Confusion between contiguity and causality (Hume, 2008; Puddu, 2000)
- Confirmation bias or verificationism (Bacon, 1620; Popper, 1959; Jones and Harris, 1967; Jones and Nisbett, 1971; Lewicka, 1998) (**Figure 1**; **Table 1**, Attribution errors).

**Table 1:** Correlation attribution errors.

		1	2	3	4	5	6	7	8	9	10
1	Fundamental attribution error	1									
2	Correspondent inference	–0,008	1								
3	Constancy	0,128	–0,092								
4	Tautology	–0,038	0,432^∗∗^	–0,053	1						
5	Norm of internality	0,070	0,232	0,200	0,040	1					
6	Attribution to the victim	0,083	–0,020	0,334^∗^	0,045	0,000	1				
7	Illusory correlation	0,179	0,092	0,292^∗^	–0,061	–0,005	0,206	1			
8	Confirmation bias or verificationism	0,163	0,216	0,370^∗^	0,055	0,501^∗∗^	0,100	0,362^∗^	1		
9	Confusion between contiguity and causality	0,216	0,300^∗^	0,117	0,109	0,157	–0,030	0,190	0,019	1	
10	TOT_Attribution errors	0,416^∗∗^	0,520^∗∗^	0,520^∗∗^	0,369^∗^	0,554^∗∗^	0,382^∗∗^	0,449^∗∗^	0,671^∗∗^	0,431^∗∗^	1

**FIGURE 2:**
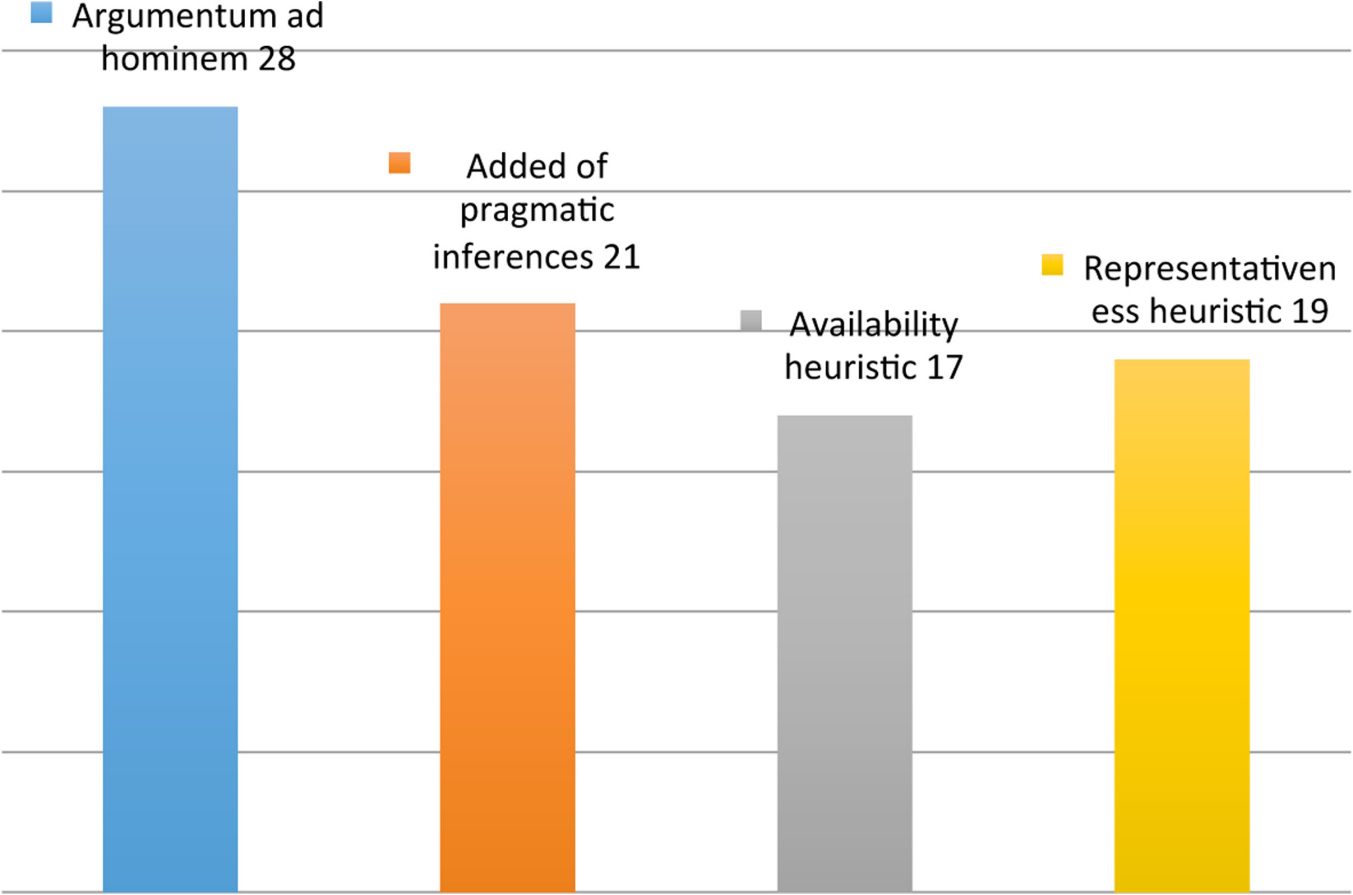
**Inferential errors**.

(b) The area of the inferential errors, that is those cognitive strategies that can carry to the construction of false nexuses of causality (like the heuristic of the availability);

- Argumentum ad hominem (Locke, 1690; McDougall, 1915; Hamblin, 1970; Woods and Walton, 1972, 1982; Copi, 1986; Douglas, 2008)
- Added of pragmatic inferences (Gazdar, 1979; Levinson, 1983; Johnson-Laird, 1983; Johnson-Laird and Byrne, 1991; Sperber and Wilson, 2002; Wilson and Sperber, 2003)
- Availability heuristic (Tversky and Kahneman, 1974)
- Representativeness heuristic (Kahneman and Tversky, 1972; Tversky and Kahneman, 1974) (**Figure 2**; **Table 2**, Inferential errors).

**Table 2:** Correlation inferential errors.

		1	2	3	4	5
1	Argumentum ad hominem	1				
2	Added of pragmatic inferencies	0,220	1			
3	Availability heuristic	0,302ˆ*	0,069	1		
4	Rapresentativeness heuristic	-0,058	-0,166	0,048	1	
5	TOT_inferential errors	0,702ˆ**	0,485ˆ**	0,602ˆ**	0,401ˆ**	1

**FIGURE 3:**
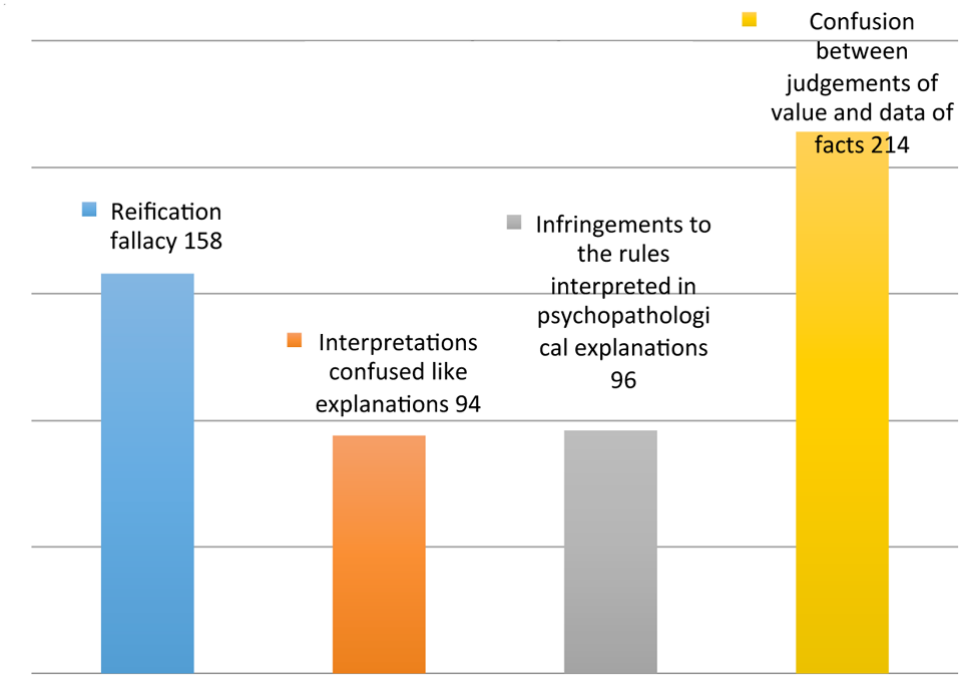
**Epistemological errors**.

(c) The area of epistemological inconsistencies that occur when the consistency between the theoretical assumptions is not respected, the tools used and the definition of the object of investigation, for example where a descriptive expedient is treated and measured as if it really existed as an empirical object.

- Reification fallacy (also known as concretism, or the fallacy of misplaced concreteness) (Whitehead, 1919, 1925; Turner and Edgley, 1980; Jablensky, 1999; Salvini, 2004)
- Interpretations confused like explanations (MacCoun, 1998; Salvini, 2004)
- Infringements to the rules interpreted in psychopathological explanation (Foucault, 1969; Quadrio and De Leo, 1995; Salvini, 2004)
- Confusion between judgments of value and data of fact (Weber, 1922; Nagel, 1961; Scriven, 1974; Plous, 1993; Iudici and De Aloe, 2007) (**Figure 3**; **Table 3**, Epistemologic errors).

**Table 3:** Correlation epistemological errors.

		1	2	3	4	5
1	Reification fallacy	1				
2	Interpretation confused like explanation	–0,011	1			
3	Infringements to the rules interpreted in psychopatological explanation	–0,108	–0,147	1		
4	Confusion between judgements of value and data of fact	–0,037	–0,425^∗∗^	0,095	1	
5	TOT_Epistemological errors	0,601^∗∗^	0,090	0,411^∗∗^	0,508^∗∗^	1

**FIGURE 4:**
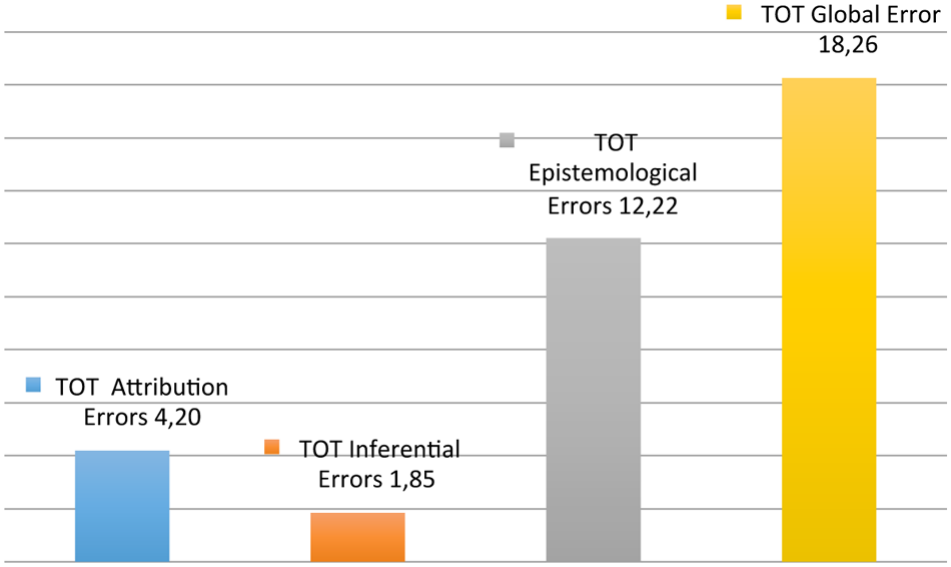
**Average number of errors per category**.

Regarding the second objective (see Appendix 2) 840 errors were identified: 193 attribution errors (22.98%), 85 inferential errors (10%) and 562 epistemological errors (66.9%) (**Figure 4**; **Table 4**, Average number of errors por category).

**Table 4:** Correlation global errors.

		1	2	3	4
1	TOT_Attribution errors	1			
2	TOT_Inferential errors	–0,171	1		
3	TOT_Epistemological errors	0,255	–0,074	1	
4	TOT_Global error	0,717^∗∗^	0,151	0,793^∗∗^	1

*^∗∗^The correlation is significant at the 0.01 level (2-tailed).*

*^∗^The correlation is significant at the 0.05 level (2-tailed).*

For the first identified area, the types of errors that appear to be committed more frequently are the error of fundamental attribution (28/193), the internality norm (28/193) and the auto confirmation tendency (27/193).

For the second area, the errors that are more often committed are the argumentum ad hominem (28/85) and the addition of pragmatic inferences (21/85). For the third area, the most frequent errors are reification (158/562) and confusion between value judgments and factual data (214/562), areas that may contain errors with higher frequencies.

### Data Analysis

Data analysis was performed using the statistical software SPSS (Statistical Package for Social Sciences [SPSS], 2008) containing procedures for exploration, management, modeling, and analysis of a data set. This tool allows to perform basic statistical analysis (description and data screening, analysis of the difference of means, non-parametric statistics), multivariable statistical analysis (multiple linear regression, factor analysis, variance analysis, discriminant analysis, multivariable analysis of variance, cluster analysis) and categorical data analysis (logistic regression models log linear). The techniques that can be used are commonly applicable in contexts defined as quantitative and qualitative. These techniques of data analysis are based on sophisticated and complex mathematical-statistical models (Barbaranelli, 2006). An appropriate statistical was calculated (linear correlation coefficient of Pearson) to evaluate the existence of significant relationships between the variables of interest: errors of attribution, inferential errors, mistakes, and epistemological between the three areas and the ‘global error (Barbaranelli and D’Olimpio, 2007). The Pearson coefficient is used to measure the correlation between variables at intervals or equivalent reports. It is the sum of the products of the standardized scores of the two variables (zxzy) divided by the number of subjects or observations. This coefficient can take values ranging from –1.00 (between the two variables there is a perfect correlation negative) and +1.00 (between the two variables there is a perfect correlation positive). A correlation of 0 indicates that between the two variables there is no relationship. In the first table, correlations between errors related to the errors of attribution are identified. You can see significant positive correlations between the error and the corresponding inference tautology (*N* = 46, *r* = 0.432, *p* < 0.05, two-tailed) and between the norm of internality and the tendency to self-confirmation (*N* = 46, *r* = 0.501, *p* < 0.05, 2-tailed), for which the variation of the first error also tends to vary that associated. More significant positive correlations are found between the error of the texture and the attribution to the victim (*N* = 46, *r* = 0.334, *p* < 0.01, two-tailed), the illusory correlation (*N* = 46, *r* = 0.292, *p* < 0.01, two-tailed) and the tendency of autoconfirmation (*N* = 46, *r* = 0.370, *p* < 0.01, two-tailed), and between the latter and the illusory correlation (*N* = 46, *r* = 0.362, *p* < 0.01, two-tailed). All errors are found to have a significant impact on the total number of errors committed in relation to the first outlined area, particularly those of the tendency to autoconfirm (*N* = 46, *r* = 0.671, *p* < 0.05, two-tailed) and the internality norm (*N* = 46, *r* = 0.554, *p* < 0.05, two-tailed).

In the second table, correlations between inferential errors have been identified. You can see the significant positive correlation between argumentum ad hominem errors and the heuristic availability (*N* = 46, *r* = 0.302, *p* < 0.01, two-tailed) for which the variation of one of the two errors also tends to vary the other. All errors are found to have a significant impact on the total number of errors committed in relation to the second outlined area, particularly argumentum ad hominem (*N* = 46, *r* = 0.702, *p* < 0.05, two-tailed) and heuristic availability (*N* = 46, *r* = 0.602, *p* < 0.05, two-tailed).

In the third table, correlations between epistemological errors are identified. You may notice in this case a significant negative correlation between the errors of interpretation argued as explanations and confusion between value judgments and factual data (*N* = 46, *r* = –0.425, *p* < 0.05, two-tailed) to indicate that with increasing of the one, the other tends to decrease or to be absent. The error of the interpretations reasoned as explanations is the only area not to have a significant influence on the total of epistemological errors committed, while the heavier weights come from the error of reification (*N* = 46, *r* = 0.601, *p* < 0.05, two-tailed) and the confusion between value judgments and factual data (*N* = 46, *r* = 0.508, *p* < 0.05, two-tailed).

In the fourth table, correlations between the total errors for each area, were found to have a significant positive about the overall weight of the total errors of attribution (*N* = 46, *r* = 0.717, *p* < 0.05, 2-tailed) and the total of epistemological errors (*N* = 46, *r* = 0.793, *p* < 0.05, two-tailed) and not the total number of errors resulting inferential unrelated.

## Discussion

Regarding the frequency of errors, you notice that the epistemological error to exchange a fact for a value judgment is present in all the expert reports considered and is most frequently committed (214 errors of 840). Well represented are also the error of reification, present in 43 surveys (158 errors on 840), the mistake to argue interpretations as if they were facts, present in 35 expert explanations (94 errors over 840) and the error of understanding the offense or the rules in terms of psychological explanation in 32 surveys (96 errors on 840). Ultimately, epistemological errors are the most common. As for inferential errors and errors of attribution, the data table shows for the first, a greater presence of argumentum ad hominem, present in 20 surveys (28 errors out of 840) and the addition of pragmatic inferences in 17 surveys (21 mistakes to 840) and, for the latter, the fundamental attribution error, present in 20 surveys (28 errors on 840), the norm of internality in 19 surveys (28 errors over 840) and the trend in this auto confirmation, present in 18 surveys (27 errors on 840). In terms of absolute frequency, the error that appears most is treating value judgments as matters of fact, belonging to the epistemological errors that may contain errors with higher frequencies. Regarding the correlations between the errors for each area and in compliance with the global error, you may notice some significant relationships. As for the area of the errors of attribution, you find that the variation of the tautological errors also varies corresponding to the inference and vice versa (*p* < 0.05). This may indicate the positive association between the explanation of behavior based on psychological traits by which you identify the same, and the use of pathological traits of personality or psychological causes or psychopathological to explain socially disapproved gestures. It is further noted that the variation of the internalities rule also tends to vary the auto confirmation trend (*p* < 0.05). This may indicate the positive association between holding a self-determined person responsible regardless of his conditions, and selecting the information in order to confirm their beliefs or belittle those that contradict them. Other significant correlations emerge (*p* < 0.05), for example one for which the variation of the tendency of auto confirmation also tends to vary the error of constancy. In this case, the association could be with the selection of information described and the prediction of persistence or re-enactment of behavior, because they “caused” by a stretch of a personality disorder. Finally, the strongest point over the total errors of the first area concerns the norm of internality and tendency of auto confirmation, most frequent errors together with the fundamental attribution error. Compared to the second area of the errors, inferential, you find that the variation of argumentum ad hominem also tends to vary the availability heuristic (*p* < 0.01). This may indicate a relationship between the information considered reliable of another person, compared to that of the protagonist because of the attitude that the preliminary estimate of the possibility that a certain event is present, or has been present, or may be experienced on a knowledge base-expectations-questions “available” at the time. These two errors appear to be those with greater weight on the total inferential errors. In the third area of epistemological errors, the greatest weight is given by the errors of reification and the confusion between value judgments and factual data, the most frequent errors in the area and in general. There is a significant negative correlation (*p* < 0.05) between the exchange of value judgments in factual data and interpretation reasoned as an explanation, so the variation of one tends to vary the other in the opposite direction. In particular, it is noted that as the first increases, the second decreases of tends to be absent. This may indicate that the higher the valuations based on a system of values or common sense presented as objective assessments, the lower (or absent) are the interpretations or theoretical conjectures presented as explanations or causes of what are investigated. Finally, the total attribution and epistemological errors have a significant impact (*p* < 0.05) on the overall error, unlike the total number of errors for which no inferential identifies a significant correlation.

## Conclusion

This study highlights how the “systematic errors of judgment,” usually referring to common sense and the man in the street, are sometimes present even in the forensic evaluation by specialist consultants. In all expert reports analyzed, several errors were identified, attributable to the following three areas: attribution errors, inferential errors, and epistemological errors.

The research highlights a significant amount of epistemological errors, as well as allocation and inferential errors. The fact that the inference attributional errors are present in much lesser extent, can perhaps be explained by the increased attention that has been paid to these in academic research. In reference to the epistemological errors, this data concurs with what has already been stated by several authors (Gulotta, 2000; Salvini, 2004), who argue that such errors can be created when psychologists make their own categories or legal terms – that have no scientific evidence in psychology – without making a “proper translation” (see for example “the ability of discernment,” “infirmity or mental disorder”). For example, when, in the evaluation for the purpose of “attribution,” it resorts to tautological conceptualizations of psychiatric nosology which is rich (see “reaction to short circuit,” “abnormal reaction,” “irresistible impulse”). From pondering on the results, the need to problematize the questions posed by the client legal emerges, because in some cases the demand that the law addresses the psychology can induce the psychologist to seek causal relations, to apportion blame and to identify solutions that psychology cannot always offer. Hence, the risk of falling into the errors presented. A further point of reflection concerns the adequacy of psychological theories to which the consultant refers to the context in which they are used: in fact, if some theories can be functional to troubleshoot clients in the clinical context, the same theories are not necessarily effective in the evaluation of people in the legal field.

In conclusion and beginning from how much emerged from the work presented, some indications can be made operational, in particular:

(a) in the formation field, to raise awareness of the scientists of the psyche who approach the forensic field to monitor their cognitive processes, include the possibility to incur errors of reasoning;
(b) in the interdisciplinary relationship, the ability to problematize the legal questions raised by the client, because it is the question that defines and limits the reality in which the consultant is called to work, so if the psychologist or psychiatrist must move on paths defined by law, it is possible that in order to answer a question as it is formulated it may cause some errors that could invalidate the entire technical-scientific procedure;
(c) the opportunity to make known the implications of some cognitive processes related to technical advice, not only in who is appointed to carry it out or who is in training, but also for those who will use the results, namely the lawyers and judges.

## Conflict of Interest Statement

The authors declare that the research was conducted in the absence of any commercial or financial relationships that could be construed as a potential conflict of interest.
