# Supplementary material for: The Clinical Assessment in the Legal Field: An Empirical Study of Bias and Limitations in Forensic Expertise
Source: Front Psychol. 2015 Nov 30;6:1831. doi: 10.3389/fpsyg.2015.01831 (PMC4663720; doi:10.3389/fpsyg.2015.01831)
Supplement: Supplementary file 1 [file Data_Sheet_1.DOCX]

***SUPPLEMENTARY DATA***

**APPENDIX 1**

**THEORETICAL DESCRIPTION ERRORS**

AREA 1

**FUNDAMENTAL ATTRIBUTION ERROR**

Historically identified by Heider (1958), in which the "cause" of behavior, whether it is in the role of observers, is attributed to a characteristic of the person (section, type, class, gender, membership, etc.) and under-estimates the intentions in relation to the situation. The fundamental attribution error is most visible when people explain the behavior of others. It does not explain interpretations of one's own behavior—where situational factors are often taken into consideration. This discrepancy is called the actor–observer bias.

Example:

*"The ritual of concealment of corpses and replacement of licence plates is explained by the narcissistic dimension of the subject."*

**CORRESPONDENT INFERENCE**

Where there is an inference of pathological personality traits or psychological or psychopathological causes beginning from the explanation of behaviors or gestures that are socially disapproved (post hoc explanation).

Example*:*

*"Aim to a realization of every aspect of his thrusts/ instinctual drives, in contempt feelings of others and of society and the world with its social and ethical rules, and the need to adapt, at least some of these rules. It follows that there is a personality structure oscillating between the histrionic and narcissistic";*

CONSTANCY

Where you make a prediction of constancy with respect to a behavior or consider that it will still be in existence because it is “caused” by a personality trait or personality disorder. This is a stereotypical generalization "based on the presumption that certain conduct is attributable to a single cause, such as when it was held that" drug users have also had a family life where the mother is or was too present and the father absent ". Generalizing is looking in the direction of the family life of each drug addict in search of predisposing factors. The "constant impressions" of the personality traits attributed are favored from the invariance of the physical aspect, from the stability of the roles and the report of the context (for example diagnostic and therapeutic), and from the impossibility to invalidate the polysemy, polyvalence and indeterminacy of psychodiagnostic terms used. Other researchers have shown that if accessibility, presence and retrieval mnemic, the vocabulary of a clinician, it will partially determine its use.

Example:

*"There is a sure negative family humus in the fatherly figure characterized by a strong violent connotation. This has determined the structuring of the character to confirmation of the rule for which if his parent is alcoholic his son will be drug addicted”.*

TAUTOLOGY

The tautology is when a behavior defined by a rule or a violated rule, after being identified as a psychological trait, is used in explanatory terms to explain that same behavior.

For example stating that "anti-social behavior, brawling and immoral to B., who is accompanied by sadistic impulses in the aims, and complacent in the effects, besides deprived of feelings of guilt, is a psychopathic behavior," *adding that "the cause of behavior of B. is due to its psychopathy*".

Example*: "It seems I handed in a best light and more brighter than that real, therefore, it's histrionic personality disorder."*

**NORM OF INTERNALITY**

Where the person is deemed capable of self-determination and individual responsibility for what he did, regardless of his condition.

Example:

*“A psychotic, a serious schizophrenic, doesn’t necessarily have the inability to intend and to want at the moment of the act because such act can be "within their reach”, even if illegal, they, to fall back on, in a situation of inability to understand and will most situations of life ".*

**ATTRIBUTION TO THE VICTIM**

When the victim is considered responsible for what happens to them or for their disadvantaged condition or is devaluated by the consequences of what happened.

Example:

*"There is no doubt that some of his misadventures, of the it is sought".*

ILLUSORY CORRELATION

Where a relationship is detected between variables that do not really have a relationship among them, neither cause-effect, but are randomly related, such as biographical adverse events and deviant behavior.

Example:

*"We find genesis of crime in a tragic series of family misfortunes, refusals, abandonments, of coming and going from founding, of hospitalizations, of dramas, shelters, dramas, not last the rape”.*

CONFUSION BETWEEN CONTIGUITY AND CAUSALITY

Where a cause-effect relation is attributed between two events, only on the base of their temporal succession.

Example:

*“The anamnestic data show us like all that was accentuated from the whirl that after the 16 years was caused from the considerable trauma that resulted in itself a long and painful iter of refuges and interventions that have invaded the late-adolescent age and that they have created a closed personality, narcissistic, detached, dominated from the withdrawal”.*

**CONFIRMATION BIAS OR** VERIFICATIONISM

When whatever consideration, agreeing or divergent, regarding the hypothesis, is used in order to confirm it, and where the contradiction is ignored.

Example:

*“the many responses indicate then a great heritage of empathy, affection, ability to identification available to the ego (other is then if of such property’ is done or is doing waste)”*

***AREA 2***

**ARGUMENTUM AD HOMINEM**

When exists a preliminary attitude of distrust or of trust towards the assertions of a person (is sick and is not aware, or says, "regretted" the truth), considering reliable instead those of a third person attributed to by the valuer.

Example:

*"He says, it sustains that the conflict with the guardians of the order that now would have been attenuated… difficult to know if this is the truth, we can only hope, and hope that the "have made ​​progress," which applies to a volunteer assistant ".*

**ADDED PRAGMATIC INFERENCES**

When there is an addition of new information to the premises, but on the base of own acquaintance of the world.

Example:

*“Obviously, people without a consenting partner use the services of prostitutes”.*

**AVAILABILITY HEURISTIC**

Where we estimate the chance that an event is present, or has been, or will occur, based on a knowledge-expectations-questions "available" at that time.

In the legal-forensic field, this distortion is realized conjugating the crime (that it is a legal construct) with the factual realism of the psychodiagnostic (than instead constructs are expressed in a mentalistic and conventional language) and with the psychic characters attributed to the offender and therefore with the availability of preordained enunciated linguistic. In the search for a relation between the personality and the trouble-offense, availability heuristic makes usable and confirmatory l' effect already mentioned of the "post hoc therefore propter hoc": evaluative and diagnostician statement conducts in the logical fallacy of the "after and therefore to cause"."In this case, the diagnostic disciplines facilitating the illusion that they can judicially construct) and personality (psychological construct), between crime and "mental illness" (analogical construct). Or establishing a causal relationship between psychobiographical elements, personality, deviance and crime through a process "post hoc antecedent": logical procedure that can be a source of deductive errors on the methodological plan, both by the experts as in the investigating magistrates, as by the judges.

Example:

*"The lexicon is good, but artificial and not correspondent to its cultural level";*

**REPRESENTATIVENESS HEURISTIC**

A form of reasoning in which the judgment is formulated based on prototypical assumptions or stereotypes in order to argue its own thesis.

Example:

*"There will always be able to stop at the right time, and certainly is able to transcend the tragedy is typical of his sadistic paraphilia in which one knows where it starts but not where it ends."*

***AREA 3***

**REIFICATION FALLACY** (also known as **concretism**, or **the fallacy of misplaced concreteness)**

When some categories belonging to the lexicon of the observer (how adjectives, judgments of value, enunciated interpretative) are translated in mental "things", in "psychic entity" really existing, as the characteristics (stable) attributed to the way the person is and to their nature.

Since the constructs and semantic interpretation cannot be logically applied to the connecting "cause and effect", the reification (and also the enunciated literalization) is the expedient to turn a linguistic construct, for example the adjective attributed to a behavior, in an entity, a property in the psyche of the person. The psychic entity so reified, that it is transformed into a "thing", making it likely to be thought of as a real "cause" and not a construct of observation, however, considering scientific factual sense.

Example:

*“What emerges is a personality intellectually gifted, but very immature with deep needs of dependence from a maternal figure and mechanisms of negation of this need ";*

**INTERPRETATIONS CONFUSED WITH EXPLANATIONS**

Where a conjecture is confused with the explanation, with a known cause of what you want to explain.

Example:

*“The narrow maternal fusional from a side facilitated from the beginning every thing, and the impossible one made possible, but at the same time it constituted a deadly and suffocating embrace that produced the archaic reaction of destructive fury, connected with the pleasure, pleasure arising from the revenge and the pain of the victim in that moment how much never near”;*

**INFRINGEMENTS TO THE RULES INTERPRETED IN PSYCHOPATHOLOGICAL EXPLANATION**

Where many authors (Foucault, 1969, Quadrio, De Leo, 1995) from various disciplines have shown the weakness of categorical reasoning that turns good and bad into normal and pathological. Where the offense and the moral transgression of social norms have been transformed into a psychopathology, to explain the deviant behavior.

Example:

*“We touch here the true, psychopathological and psychoanalytic sense, of the term of perversion, in the sense properly of the erotic pulsione, that is aimed towards pleasure, which changes sign at hand, at the time of realization, from positive to negative, of the obtainment of the pleasure from the situations that they create pain and disgust";*

**CONFUSION BETWEEN JUDGMENTS OF VALUE AND DATA OF FACT**

When an assessment based on a system of values or common sense is presented as an objective evaluation, considered as an incontrovertible fact and omitting and not clarifying implications of value contained in it. It therefore attributes a valence of moral judgment to enunciated psychological, retroactive or predictive type, or gives scientific importance to moralizing opinions and comments.

Example:

*“They are incapable to join to ironic bickering of colleagues” (DSM IV-TR, p. 743); or "there is a redoubt ability to feel pleasure in sensory experiences such as walking on the beach at sunset or to make sex" (DSM IV-TR, pag.739);*

**APPENDIX 2**

**Survey Resulted Error**

We describe below some examples of the errors in the expert reports examined in this research

**FUNDAMENTAL ATTRIBUTION ERROR**

*“A component histrionic... a narcissistic approach, in which prevails the inner reality ... in this sense it is understandable that communication, in its entirety, become poor, limited, without the accompaniment of liveliness and emotional, although the specific and targeted questions reveal a specific expertise to answer, only present if stimulated, hardly autonomous, to witness the size more histrionic that narcissistic of its mode of relationship” (Expert Reports n.25)*

**CORRESPONDENT INFERENCE**

*"She committed suicide because it had a personality trait depressed"* *(Expert Reports n.33)*

*“The man who committed the violent act is affected from serious personality disorder in which emerged traits borderline, paranoid, antisocial, narcissistic and sadistic*” *(Expert Reports n.23)*

CONSTANCY

*“The Delirious Trouble is not accidental or transient, so the psychiatric judgment is of social danger";*

*(Expert Reports n.20)*

TAUTOLOGY

*"Since the point of these situations is always that of I handed in a best light and brighter than the real one, if you want to fix this psychopathology, one can only resort to histrionic personality disorder. It is personality with a pervasive and excessive emotionality aiming to the approval or to the search of acceptance and attention. Often to get the result, can "dramatize" that is put on stage something, inventing a script, lie, tell stories (fantastic pseudologia) to put themselves on the attention needed or desired place himself in the situation". (Expert Reports n.40)*

**NORM OF INTERNALITY**

Example:

"It's a drug addict because he wanted to become that" *(Expert Reports n.45)*

"Even if it is schizophrenic is free to self-determination, and is imputable»  *(Expert Reports n.10)*

**ATTRIBUTION TO THE VICTIM**

*Example:*

The aggressor needs an outlet for his sexual aggressiveness and finds a submissive partner who unconsciously invited him to sexual abuse in order to satisfy his masochistic needs

*(Expert Reports n.04)*

ILLUSORY CORRELATION

Example:

A long and painful iter of hospitalizations and interventions. they have created a closed personality, narcissistic, detached, dominated by the withdrawal" *(Expert Reports n.14)*

**CONFIRMATION BIAS OR** VERIFICATIONISM

*Example:*

*“The split of the conscience of the ego goes reported to the category nosological of the Dissociative Troubles. ... in our case would then be the Dissociative Identity Disorder ... in our case, however, the excessive repetition, the precision and structuring behavior, psychomotor function sequences , the integration of all activities into a unified personality, well, they rule out a framework of this kind. It remains only amnesia in support of specific behaviors, but that is a posteriori and dissimulated, or, if you want to include in the functional disorders of memory and consciousness of the ego, rather referring to expressions of type conversion ganseriano”.*

*(Expert Reports n.30)*

*“Depression is not found, but exists of fund, submerged from the casual and grandiose performance”.*

*(Expert Reports n.17)*

*“Of the violences of the father however it is not complained … difficult to say if … the problem arises with the difficulty -even if denied- with the father figure";”*

*(Expert Reports n.42)*

*“Has it shown to include fully the meaning of the questions to him revolted, with always pertinent replies, but the assertions, simply pertinent, can appear indicative of a condition of the subject significantly altered in psychopathological sense”*

*"Disharmony is the greatest show the expected second-in-reactivity, which emerges at the slightest hint of disagreement ...". (Expert Reports n.13)*

CONFUSION BETWEEN CONTIGUITY AND CAUSALITY

Example:

“The successful separation led to isolation and depression clinically significant”  *(Expert Reports n.39)*

**ARGUMENTUM AD HOMINEM**

Example:

“At least according to what she says " *(Expert Reports n.19)*

"Was a manifestation of violent reactivity to the wise words of the assistant voluntary '

*(Expert Reports n.21)*

**ADDED OF PRAGMATIC INFERENCES**

Example:

“It is well known that drug addicts are vulnerable people” *(Expert Reports n.26)*

*How much declaring could not be true since of forehead we have a histrionic personality (Expert Reports n.4)*

**AVAILABILITY HEURISTIC**

Example:

“Those who deal of violence easily recognizes that this is an aggressive person”  *(Expert Reports n.09)*

*“*And well known that today separations are very common*” (Expert Reports n.18)*

**REPRESENTATIVENESS HEURISTIC**

Example:

*“Such behavior is typical of someone who has a damaged relationship with reality” (Expert Reports n.07)*

*“Mr. X does not understand how a mother can suffer if there is no custody of her son” (Expert Reports n.38)*

**REIFICATION FALLACY** (also known as **concretism**, or **the fallacy of misplaced concreteness)**

Example:

*“An excessive reception of the stimulus, from the personality”; (Expert Reports n.33)*

*“Doing not work the preconscious with his capacity, it is given back possible alone the acting to put in function and to concretize the inclined pulsionalis that for him they took only existence when they realized him in facts and behaviors”; (Expert Reports n.44)*

*“The subject is the bearer of pyromania, is not responsible for the fires started, because the disease takes the place of free will”; (Expert Reports n.15)*

*"the sexual sadism, alone, without interventions of mental disorders, can conduct to the same facts, in coordinated way and structured";(Expert Reports n.31)*

*“Once handcuffed, in order to unload the strong aggressive charge, has begun to give heads against the cowling of the car”. (Expert Reports n.37)*

**INTERPRETATIONS CONFUSED LIKE EXPLANATIONS**

Example:

“*The experience of the child has produced] a deep resentment over the abandonment implied in the former situation, with a great need for compensation for narcissistic for the great suffered frustration, revanche that is expressed in the direction taken by libidico-emotional drive of control and total dominion on the female figure, the origin of every abandonment: sadistic control has the function to realize the pleasure to him denied in the childish demands, with the fantasy that to this revanche edipica is associated the guarantee not to be abandoned for the total control of the love-hated object, in an omnipotent narcissistic ambivalence”; (Expert Reports n.19)*

*“She has consistently maintained relationships with several men from whom was humiliated because she is masochistic"; (Expert Reports n.29)*

*“There was a framework of general alarm, which affects the coenaesthesia, the distrust in his own body, the sense of general precariousness and the need of confirmation and reassurance. On this kind of inertia, generically psychophobic, the great dependancy of the patient, that it probably produced a series of confirmation demands through the control of the people, essentially of the partners”; (Expert Reports n.43)*

*“Inside this archaic relationship, regressive, of love and hate, it is developed the deadly embrace, with the formation of a Super Io archaic and of an ideal unattainable of the self, for which, in the impossibility to produce the social performances and of study demanded from the mother, it is outlined the sole possible solution, the pathological lie"). (Expert Reports n.40)*

**INFRINGEMENTS TO THE RULES INTERPRETED IN PSYCHOPATHOLOGICAL EXPLANATION**

Example:

*“They are frequently deceitful and manipulative for profit or personal enjoyment"; (Expert Reports n.18)*

*"All his sex life, but we can say his whole mental life, is rotated around these abnormal mental representations, first through scoptofile activities or publications of photos like that and then , over time, around designs of rituals sadists”. (Expert Reports n.36)*

**CONFUSION BETWEEN JUDGMENTS OF VALUE AND DATA OF FACT**

Example:

*“The conversion and the pseudologia (pathological lying) are not so much an illness how much a style of life. You goes from the modest lie, to the folklorist mythomaniac, the braggart jovial, to the dark and left matricide”; (Expert Reports n.45)*

*“Individuals with this disorder have an excessively impressionistic speech, exaggerated expression of emotions that seem to go on and off too quickly ... Show a certain blunt end, devoid of the playful joyfulness surface mythomaniac.”; (Expert Reports n.02)*

*“He lives in a great house, isolated, old but not bare, cold, which may well represent the house of horrors” (Expert Reports n.23)*

*“The mood seems appropriate to the situation”; (Expert Reports n.01)*

*“Individuals with panic disorder may have Inflated self-esteem and may be too arrogant and stubborn, self-confident or conceited. They can have a casual appeal, superficial, and can be rather fickle and complacent”. (Expert Reports n.32)*
